# Temporal patterns of chronic disease incidence after breast cancer: a nationwide population-based cohort study

**DOI:** 10.1038/s41598-022-09542-w

**Published:** 2022-03-31

**Authors:** Danbee Kang, Minwoong Kang, Yun Soo Hong, Jihwan Park, Jin Lee, Hwa Jeong Seo, Dong Wook Kim, Jin Seok Ahn, Yeon Hee Park, Se Kyung Lee, Dong Wook Shin, Eliseo Guallar, Juhee Cho

**Affiliations:** 1 Department of Clinical Research Design and Evaluation, SAIHST, Sungkyunkwan University, Seoul, South Korea; 2 Center for Clinical Epidemiology, Samsung Medical Center, Sungkyunkwan University, Seoul, South Korea; 3 Department of Digital Health, SAIHST, Sungkyunkwan University, Seoul, South Korea; 4 Departments of Epidemiology and Medicine, and Welch Center for Prevention, Epidemiology and Clinical Research, Johns Hopkins University Bloomberg School of Public Health, Baltimore, MD USA; 5 Medical Informatics and Health Technology (MIT), Department of Health Care Management, College of Social Science, Gachon University, Seongnam, South Korea; 6 Department of Information and Statistics, Gyeongsang National University, Jinju, South Korea; 7 Division of Hematology/Oncology, Department of Medicine, Samsung Medical Center, Sungkyunkwan University School of Medicine, Seoul, South Korea; 8 Department of Surgery, Samsung Medical Center, Sungkyunkwan University School of Medicine, Seoul, South Korea; 9 Department of Family Medicine, Samsung Medical Center, Sungkyunkwan University School of Medicine, Seoul, South Korea

**Keywords:** Cancer, Medical research, Oncology, Risk factors

## Abstract

We conducted a retrospective cohort study to evaluate the temporal pattern of incidence of chronic conditions after developing breast cancer using a population-based national registry. We selected 84,969 women with newly diagnosed breast cancer between 2002 and 2016 and a 1:10 sample of age-matched non-breast cancer controls (N = 1,057,674). The main study exposure was incident breast cancer, considered as a time-varying exposure. The outcomes were incident cases of leukemia, endometrial cancer, myeloma, cardiomyopathy, osteoporosis, end stage renal disease (ESRD), pulmonary fibrosis, hypothyroidism, type 2 diabetes, hypertension and hyperlipidemia. The development of breast cancer was associated with a significantly increased risk of all outcomes analyzed except for ESRD and hypertension. The fully-adjusted risks of leukemia (HR 3.09; 95% CI 2.11–4.51), cardiomyopathy (HR 2.65; 95% CI 1.90–3.68), endometrial cancer (HR 3.53; 95% CI 2.76–4.53), hypothyroidism (HR 1.29; 95% CI 1.19–1.40), pulmonary fibrosis (HR 1.84; 95% CI 1.12–3.02), and hyperlipidemia (HR 1.24; 95% CI 1.20–1.28) remained significantly elevated after more than 5 years since diagnosis. Optimal care for breast cancer survivors requires close collaboration between oncologists and allied health care professionals to identify and manage the long-term morbidity and mortality associated with these chronic conditions.

## Introduction

Breast cancer is the most common malignancy in women, with an average lifetime risk of 1 in 8^1^. Thanks to early diagnosis and medical advances, the 5-year survival of breast cancer patients has increased from 79.2% in 1993–1995 to 93.2% in 2013–2017^2^. Due to the high incidence and high survival, breast cancer survivors represent one of the largest groups of cancer survivors. Breast cancer survivors, however, are at increased risk of developing new chronic conditions. Women with breast cancer had a higher risk of developing cardiovascular disease^3^, osteopenia, osteoporosis, and bone fractures^4^, hypothyroidism^5^ and second primary malignancies including acute myeloid leukemia, multiple myeloma, and acute lymphoblastic leukemia or lymphocytic lymphoma^6,7^.

Previous studies have not provided detailed descriptions of the temporal pattern of chronic disease incidence after breast cancer, partly due to limited sample sizes and to limited follow up times. Furthermore, prior studies were usually limited by a lack of information on risk factor levels prior to the development of breast cancer, making it difficult to separate the specific contribution of breast cancer or breast cancer-related therapies to the development of chronic disease^8^.

Understanding the pattern of chronic disease incidence after breast cancer can help plan preventive services and clinical management of cancer survivors, and may provide leads into the etiology of chronic disease in this patient group. We thus studied a large cohort of women without a history of cancer at baseline to evaluate the temporal pattern of incidence of chronic diseases after developing breast cancer.

## Results

**Figure 1:**
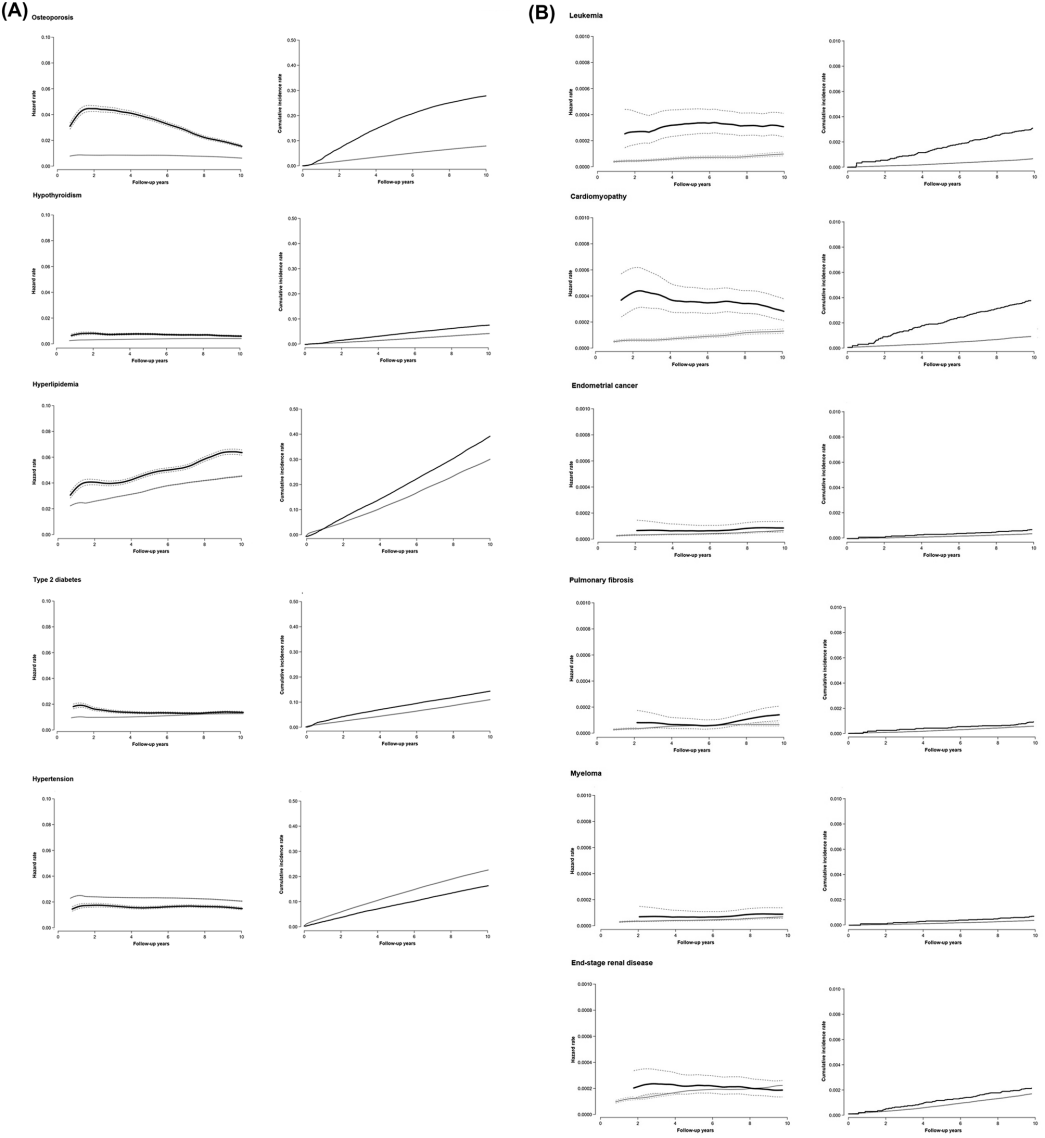
Hazard rate and cumulative incidence of (**A**) common chronic conditions and (**B**) relatively rare chronic conditions by breast cancer status. Hazard rate was calculated using a weighted kernel-density estimate using the estimated hazard contributions. Cumulative incidence was calculated using Kaplan–Meier curves. Participants who developed breast cancer contributed person-time to the exposed group from the time of breast cancer development. Unexposed person-time was contributed by participants who did not develop breast cancer and by participants who developed breast cancer prior to each comorbidity development. To reduce the potential impact of surveillance bias, we considered that outcomes occurring in the first 31 days after a diagnosis of breast cancer corresponded to unexposed person-time.

Compared to participants who developed breast cancer (N = 84,969; 425,132 person-years [py] of follow-up), non-breast cancer controls (N = 1,057,674; 10,654,144 py of follow-up) had similar characteristics at the time of the baseline health screening exam (Table 1). The development of chronic conditions was common in the study population. The incidence of study endpoints was particularly high for hyperlipidemia (overall incident cases, N = 308,095; incidence rate, 3513 per 100,000 person-years [py]), hypertension (N = 199,015; 2396 per 100,000 py), type 2 diabetes (N = 109,375; 1112 per 100,000 py), osteoporosis (overall N = 83,192; incidence rate 850 per 100,000 py), and hypothyroidism (N = 46,013; 432 per 100,000 py) (Table 2, Fig. 1). The 10-year cumulative incidence for each of the other endpoints was < 1%.

**Table 1:** Characteristics of study participants at the baseline health screening exam (N = 1,142,643).

Characteristics	No breast cancer (N = 1,057,674)	Incident breast cancer (N = 84,969)
Age at baseline, year	46 (40–54)	46 (40–53)
**Age at menarche**		
< 10 years	2660 (0.3)	394 (0.5)
10–13 years	73,185 (6.9)	7025 (8.3)
14–16 years	482,512 (45.6)	41,278 (48.6)
≥ 17 years	398,988 (37.7)	27,696 (32.6)
Unknown	100,329 (9.5)	8578 (10.1)
**Menopause status**		
Pre-menopause	404,672 (38.3)	30,291 (35.7)
Post-menopause	556,829 (52.7)	46,501 (54.7)
Unknown	96,163 (9.1)	8177 (9.6)
**BMI, kg/m**^**2**^		
Underweight (< 18 kg/m^2^)	38,847 (3.7)	3035 (3.6)
Normal (18–< 23 kg/m^2^)	474,009 (44.8)	37,724 (44.4)
Overweight (23–< 25 kg/m^2^)	243,220 (23.0)	19,577 (23.0)
Obese (≥ 25 kg/m^2^)	301,127 (28.5)	24,604 (29.0)
Unknown	471 (0.0)	29 (0.0)
**Alcohol amount**		
None	713,524 (67.5)	58,034 (68.3)
Moderate (1–19 g/day)	275,882 (26.1)	21,469 (25.3)
Heavy (≥ 20 g/day)	20,848 (2.0)	1497 (1.8)
Unknown	47,420 (4.5)	3978 (4.7)
**Smoking status**		
Never smoker	946,369 (89.5)	75,647 (89.0)
Ever smoker	58,619 (5.5)	4767 (5.6)
Unknown	52,686 (5.0)	4555 (5.4)
**Moderate-vigorous physical activity**		
None	610,131 (57.7)	48,480 (57.1)
1–2 times per week	204,126 (19.3)	16,719 (19.7)
≥ 3 times per week	195,661 (18.5)	15,701 (18.5)
Unknown	47,756 (4.5)	4069 (4.8)
**Income percentile**		
Medical aid	19,753 (1.9)	1480 (1.7)
≤ 30th	304,541 (28.8)	23,692 (27.9)
31th–70th	357,873 (33.8)	28,465 (33.5)
> 70th	375,507 (35.5)	31,332 (36.9)
**Comorbidities at baseline**		
Cardiomyopathy	537 (0.1)	30 (0.0)
Osteoporosis	66,771 (6.3)	5213 (6.1)
Hypothyroidism	18,962 (1.8)	1397 (1.6)
Pulmonary fibrosis	142 (0.0)	8 (0.0)
Hyperlipidemia	82,236 (7.8)	5968 (7.0)
End-stage renal disease	527 (0.1)	46 (0.0)
Type 2 diabetes	67,156 (6.4)	48,791 (5.8)
Hypertension	155,875 (14.7)	11,853 (14.0)

Values in the table are median (interquartile range) or number (percentage).

**Table 2:** Hazard ratios (95% confidence intervals) for incident outcomes after incident breast cancer.

Outcome*	No. of cases (incidence rate per 100,000 person-years)		Hazard ratio (95% CI)	
	No breast cancer	Incident breast cancer	Crude	Adjusted
Leukemia (N = 1,142,643)	716 (6.7)	129 (30.4)	4.19 (3.47, 5.05)	4.20 (3.48, 5.07)
Cardiomyopathy (N = 1,142,076)	957 (9.0)	143 (33.7)	3.20 (2.68, 3.82)	3.28 (2.75, 3.91)
Osteoporosis (N = 1,070,659)	74,818 (789.9)	8374 (2658.1)	2.97 (2.91, 3.04)	3.00 (2.93, 3.07)
Endometrial cancer (N = 1,142,643)	1539 (14.5)	178 (42.0)	2.69 (2.30, 3.14)	2.70 (2.31, 3.16)
Hypothyroidism (N = 1,122,284)	43,119 (420.2)	2894 (724.0)	1.68 (1.62, 1.74)	1.69 (1.63, 1.76)
Pulmonary fibrosis (N = 1,142,493)	599 (5.6)	44 (10.4)	1.60 (1.18, 2.17)	1.62 (1.19, 2.20)
Myeloma (N = 1,142,643)	434 (4.1)	31 (7.3)	1.53 (1.06, 2.20)	1.54 (1.07, 2.22)
Hyperlipidemia (N = 1,054,439)	292,404 (3452.5)	15,691 (5240.3)	1.34 (1.32, 1.37)	1.37 (1.35, 1.39)
End-stage renal disease (N = 1,141,344)	1823 (17.1)	86 (20.3)	1.03 (0.83, 1.28)	1.13 (0.91, 1.40)
Type 2 diabetes (N = 1,070,608)	104,448 (1102.2)	4927 (1358.7)	1.10 (1.07, 1.13)	1.13 (1.10, 1.16)
Hypertension (N = 974,915)	193,966 (2425.0)	5049 (1648.8)	0.60 (0.58, 0.62)	0.61 (0.59, 0.63)

Each outcome was analyzed separately. Each analysis was performed among participants free of the outcome disease at baseline and adjusted for body mass index category (underweight, normal, overweight, obese, and unknown), alcohol intake (none, moderate, heavy, and unknown), physical activity (none, 1–2 times per week, ≥ 3 times per week, and unknown), smoking status (never smoker, ever smoker, and unknown), income percentile (Medical Aid, ≤ 30th, 31st–70th, > 70th percentile), as well as for the baseline presence of comorbid conditions other than the corresponding outcome.

The development of breast cancer was associated with a significantly increased risk of all outcomes analyzed except for ESRD and hypertension (Table 2). The fully-adjusted HRs associated with the development of breast cancer were especially high for the development of leukemia (HR 4.20; 95% CI 3.48–5.07), cardiomyopathy (HR 3.28; 95% CI 2.75–3.91), osteoporosis (HR 3.00; 95% CI 2.93–3.07), and endometrial cancer (HR 2.70; 95% CI 2.31–3.16). When risk was evaluated by age, the association was particularly strong for leukemia, cardiomyopathy, osteoporosis, pulmonary fibrosis, and myeloma (Supplement Table 3).

The temporal pattern of increased risk of disease endpoints after the development of breast cancer varied by endpoint (Table 3, Fig. 1). Cardiomyopathy, osteoporosis, hypothyroidism, type 2 diabetes and ESRD had initially increased HRs that decreased over time. The risks of leukemia, and endometrial cancer were elevated in the first year after the diagnosis of breast cancer and further increased over follow-up. The risks of hyperlipidemia, pulmonary fibrosis, and myeloma were elevated in most periods over follow-up, although there was not a clear temporal pattern.

**Table 3:** Hazard ratios (95% confidence intervals) for incident outcomes after incident breast cancer by time after breast cancer diagnosis.

Outcome*	No breast cancer	< 1 year after diagnosis	1–< 3 years after diagnosis	3–< 5 years after diagnosis	≥ 5 years after diagnosis
Leukemia (N = 1,142,643)	Reference	2.35 (1.36, 4.08)	5.15 (3.84, 6.90)	5.87 (4.27, 8.08)	3.09 (2.11, 4.51)
Cardiomyopathy (N = 1,142,076)	Reference	4.30 (3.01, 6.15)	3.55 (2.63, 4.79)	3.08 (2.14, 4.43)	2.65 (1.90, 3.68)
Osteoporosis (N = 1,070,659)	Reference	4.17 (4.00, 4.36)	4.71 (4.56, 4.86)	2.42 (2.30, 2.55)	0.87 (0.80, 0.93)
Endometrial cancer (N = 1,142,643)	Reference	2.07 (1.40, 3.08)	1.87 (1.35, 2.58)	3.33 (2.50, 4.42)	3.53 (2.76, 4.53)
Hypothyroidism (N = 1,122,284)	Reference	2.06 (1.91, 2.22)	2.03 (1.91, 2.16)	1.37 (1.25, 1.49)	1.29 (1.19, 1.40)
Pulmonary fibrosis (N = 1,142,493)	Reference	1.99 (1.03, 3.85)	1.77 (1.04, 3.01)	0.83 (0.34, 1.99)	1.84 (1.12, 3.02)
Myeloma (N = 1,142,643)	Reference	2.48 (1.23, 5.00)	0.88 (0.36, 2.11)	1.79 (0.89, 3.60)	1.50 (0.80, 2.81)
Hyperlipidemia (N = 1,054,439)	Reference	1.54 (1.49, 1.59)	1.46 (1.42, 1.50)	1.27 (1.22, 1.31)	1.24 (1.20, 1.28)
End-stage renal disease (N = 1,141,344)	Reference	0.86 (0.49, 1.51)	1.35 (0.95, 1.92)	0.99 (0.62, 1.60)	1.17 (0.79, 1.74)
Type 2 diabetes (N = 1,070,608)	Reference	1.67 (1.59, 1.77)	1.14 (1.08, 1.20)	0.95 (0.89, 1.01)	0.91 (0.86, 0.97)
Hypertension (N = 974,915)	Reference	0.73 (0.69, 0.78)	0.60 (0.57, 0.63)	0.52 (0.49, 0.55)	0.61 (0.58, 0.64)

Each outcome was analyzed separately. Each analysis was performed among participants free of the outcome disease at baseline and adjusted for body mass index category (underweight, normal, overweight, obese, and unknown), alcohol intake (none, moderate, heavy, and unknown), physical activity (none, 1–2 times per week, ≥ 3 times per week, and unknown), smoking status (never smoker, ever smoker, and unknown), income percentile (Medical Aid, ≤ 30th, 31st–70th, > 70th percentile), as well as for the baseline presence of comorbid conditions other than the corresponding outcome.

After more than 5 years since the initial diagnosis, the risks of leukemia (HR 3.09; 95% CI 2.11–4.51), cardiomyopathy (HR 2.65; 95% CI 1.90–3.68), endometrial cancer (HR 3.53; 95% CI 2.76–4.53), hypothyroidism (HR 1.29; 95% CI 1.19–1.40), pulmonary fibrosis (HR 1.84; 95% CI 1.12–3.02), and hyperlipidemia (HR 1.24; 95% CI 1.20–1.28) remained significantly elevated (Table 3).

The most common combinations of therapies in addition to surgery were chemotherapy and radiotherapy (N = 38,541, 45.4%), radiotherapy (N = 14,836, 17.5%), and radiotherapy, chemotherapy, and hormone therapy (N = 10,970, 12.9%) (Supplement Fig. 1). Patients, those who received chemotherapy or radiation therapy had substantially increased risk of leukemia, cardiomyopathy, osteoporosis, and endometrial cancer, and patients who received hormone therapy had substantially elevated risks of leukemia, cardiomyopathy, osteoporosis, endometrial cancer, pulmonary fibrosis, myeloma, and ESRD (Supplement Table 2).

The temporal pattern of chronic disease incidence were similar regardless of treatment modality (Supplement Table 3). Patients who received chemotherapy had increased risk for myeloma even after 5 years since initial diagnosis (HR 2.06; 95% CI 1.07–3.99) compared to those who did not develop breast cancer. The risk for ESRD remained significantly elevated for patients who had hormone therapy even after 5 years since initial diagnosis (HR 2.40; 95% CI 1.36–3.24).

## Discussion

In this large national cohort, breast cancer development was associated with an increased risk of subsequent development of multiple chronic conditions including leukemia, cardiomyopathy, osteoporosis, endometrial cancer, hypothyroidism, pulmonary fibrosis, myeloma, hyperlipidemia, and type 2 diabetes. The risk of leukemia, cardiomyopathy, osteoporosis, pulmonary fibrosis, and myeloma was much higher in patients who were younger than 50 years. The temporal pattern of disease risk after breast cancer varied outcome, but the risk remained significantly elevated after more than 5 years since diagnosis for leukemia, cardiomyopathy, endometrial cancer, hypothyroidism, pulmonary fibrosis, and hyperlipidemia.

Several pathways may contribute to an increased risk of chronic conditions after breast cancer. First, breast cancer and other chronic conditions share a number of risk factors including unhealthy diet, physical inactivity, excess adiposity, smoking, alcohol intake, and metabolic abnormalities^9–11^. Second, cancer can accelerate the ageing process, even in the absence of cancer therapy^12,13^. These effect may be particularly relevant for the development of abnormal thyroid function, osteoporosis, cardiomyopathy, pulmonary fibrosis, and second primary tumors^13,14^. Cancer patients show increased vulnerability to stress, inability to restore physiological integrity, and accelerated frailty^15^, which can also be associated with the development of chronic conditions. Third, breast cancer patients undergo aggressive treatments, such as chemotherapy and radiotherapy, with increased risks of acute and chronic side effects.

In our study, breast cancer patients had increased risk of leukemia and multiple myeloma compared to women without breast cancer. The increased risk for leukemia was evident shortly after breast cancer development and remained elevated even 5 years after diagnosis. Genetic studies have identified somatic mutations in leukemia-related genes present in tumor-infiltrating leukocytes (TILeuks) in cases of early breast cancer who underwent chemotherapy^16^. Therapy-related myeloid neoplasms (t-MNs) have also been reported in early-stage breast cancer patients treated with chemotherapy and radiation therapy^17^. In fact, breast cancer is the most frequent solid tumor linked to t-MNs, accounting for 70% of t-MNs among women^18^.

The increased risk of cardiomyopathy was also evident shortly after breast cancer development and remained elevated throughout the rest of follow-up. Cardiomyopathy is a well-known side effect of anthracyclines. Anthracyclines induce cardiomyocyte death by interacting with DNA, binding to topoisomerase IIβ^19,20^, and generating reactive oxygen species, which damage DNA, proteins, and lipids, including the mitochondrial membrane^21^. Besides chemotherapy, radiation therapy could also affect the incidence of cardiomyopathy. Although more modern radiation therapy techniques may have attenuated the risk or cardiac toxicity, the long-term safety of radiation therapy for breast cancer remains uncertain^22^.

Incident breast cancer patients had about 2 times higher risk of developing endometrial cancer during early survivorship (< 3 years after diagnosis), and this risk increased in later survivorship (> 3 year). Endometrial cancer and breast cancer share common lifestyle risk factors such as smoking, obesity, diabetes, physical inactivity, and reproductive factors such as early age at menarche, late-onset menopause, and nulliparity. The increase in risk during survivorship may be also due to long-term exposure to unopposed estrogens^23^. For example, tamoxifen promotes cell proliferation in the endometrium and migration and invasion of endometrial cancer cells^24^, which may explain an increased risk of endometrial lesions, including hyperplasia, polyps, carcinomas, and sarcoma^25^. In fact, in a meta-analysis of 20 trials of 21,457 early breast cancer patients, the risk of endometrial cancer increased by 2.4 times in the tamoxifen group compared to the control group^26^. Considering the increased risk over time, breast cancer survivors may need to be referred for endometrial cancer screening, especially patients who received hormone therapy.

The risk of hypothyroidism was evident right after development of breast cancer and the remained significantly elevated even 5 years after diagnosis regardless of treatment. In previous studies, breast cancer patients who received radiation therapy were at higher risk of developing hypothyroidism compared to individuals without cancer^27^. The thyroid gland is highly sensitive to radiation, which may damage its blood vessels and parenchymal cells, trigger autoimmune reactions, and cause fibrosis^28^. Radiation-induced thyroid disease most commonly presents as hypothyroidism^28^. However, chemotherapy or hormone therapy-related hypothyroidism in breast cancer patients, is less well understood. Further studies are needed to understand the impact of chemotherapy and hormone on the incidence of thyroid disorders in breast cancer survivors.

We also observed a sustained risk of hyperlipidemia in breast cancer survivors over time. Lipid metabolism is associated with sex hormones^29^, and the changes in lipid levels after breast cancer correlate with changes in menstruation^29^ due to chemotherapy or hormone therapy. Also, adjuvant chemotherapy was associated with significant changes in the lipid profile^30^. Aromatase inhibitors, for instance, block the conversion of androgens to estrogen and affect the lipid profile^31^. An analysis of pooled data from seven clinical trials (N = 30,023 patients) demonstrated that longer duration of aromatase inhibitor use was associated with a statistically significant increase in the odds of hypercholesterolemia as compared with tamoxifen^31^.

Several limitations need to be considered in the interpretation of our findings. First, due to the nature of the data, we did not have information on cancer stage, and we had only limited information on breast cancer treatments and disease management based on claims. As a consequence, we could not establish the precise mechanisms responsible for increased chronic disease risk. Second, patients who develop cancer have more contact with the health care system which may induce surveillance bias. However, we excluded participants with comorbidities after a baseline screening, as well as comorbidities that were identified very early after cancer development. Lastly, we were not able to evaluate the independent contribution of each treatment modality as patients commonly received treatment combinations.

In conclusion, breast cancer development was associated with an increased risk of subsequent development of multiple chronic conditions, and the risk remained significantly elevated after more than 5 years since diagnosis. Considering that these chronic diseases may have a substantial impact on the quality of life and survival of breast cancer survivors^32^, incorporation of surveillance and prevention strategies would be necessary to manage the long-term morbidity and mortality associated with these chronic conditions in breast cancer patients. Further research also is required to define optimal strategies for prevention, early detection, and management of chronic conditions after breast cancer.

## Methods

### Study population and design

We conducted a retrospective cohort study using a population-based national registry. Korea has a universal single-payer national health system, and the National Health Insurance Service (NHIS) maintains national records of all covered inpatient and outpatient visits, procedures, and prescriptions. The NHIS also includes information on risk factor levels for common chronic conditions collected in routine health screening exams supported by the national health system.

**Figure 2:**
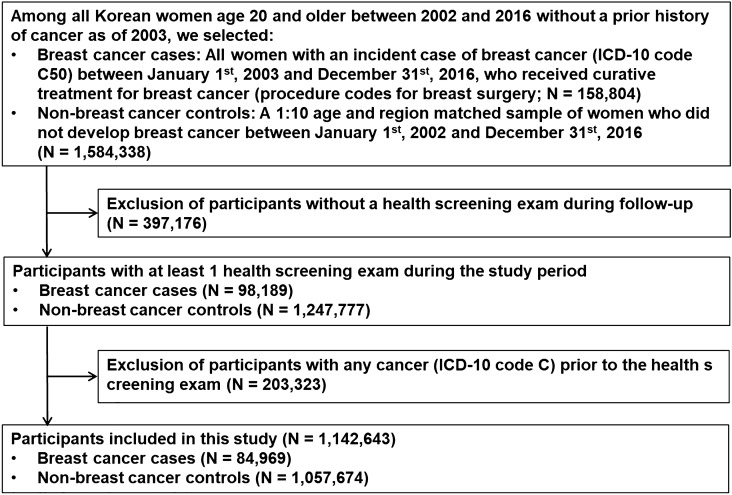
Flow chart of study participants.

Among all Korean women age 20 and older between 2002 and 2016 and without a prior history of cancer (defined as an International Classification of Diseases 10th Revision [ICD-10] C code in any prior claim) as of 2002, we selected all women with an incident case of breast cancer (ICD-10 code C50) between January 1st 2003 and December 31st, 2016 (Fig. 2). Since our study was intended to evaluate the temporal pattern of incidence of chronic diseases after developing breast cancer, we restricted our analysis to breast cancer cases who received curative treatment for breast cancer (procedure codes for breast surgery N7131 to N7135; N = 158,804). We then identified a 1:10 age and region matched sample of women who did not develop breast cancer during the study period (non-breast cancer controls; N = 1,584,338).

In Korea, the Industrial Safety and Health Law also requires annual or biennial health screening exams of all employees that include assessment of cardiovascular and diabetes risk factors. These exams are covered by the NHIS and provided free of charge. Approximately 72% of eligible beneficiaries receive the screening exams^33^. Thus, among breast cancer cases and non-breast cancer controls, we selected participants who had at least 1 health screening exam during the study period (98,189 breast cancer cases and 1,247,777 non-breast cancer controls). Among the eligible participants, we excluded 203,323 participants who had any cancer (ICD-10 code C) prior to the health screening exam as the health screening exam constitutes the baseline for the current study. The final sample size was 1,142,643 (84,969 breast cancer cases and 1,057,674 non-breast cancer controls). The Institutional Review Board (IRB) of the Samsung Medical Center approved (SMC-201912064) this study and waived the requirement for informed consent as we used only de-identified data. This study was performed in accordance with relevant guidelines/regulations of our institutions.

### Data collection

The NHIS data comprises five databases on insurance eligibility, medical treatments, medical care institutions, general health screening exams and cancer screening exam. The medical treatments database contains information on all claims on inpatient hospitalizations, outpatient visits, treatments and procedures in all clinical care use from primary care clinics to tertiary hospitals reimbursed by the NHIS, including details of diseases and prescriptions^34^. Thus, potential strengths of this data set were that we had almost all the patient with cancer as well as their follow up until they death or end date of the data set. NHIS claims for inpatient and outpatient visits, procedures, and prescriptions were coded using ICD-10 and the Korean Drug and Anatomical Therapeutic Chemical codes^35^. NHIS routinely audits the claims, and the data are considered reliable and have used in numerous peer-reviewed publications^34,36^. According to a recent study, definitions of cancer diagnosis in our study is an accurate and valid method to identify cancer incidence using health insurance claim data^37^. Definitions showed over 90% sensitivity in identifying patients with central cancer registration. For breast cancer, it had 97.9% sensitivity (95% CI, 97.8 to 98.0).

The main study exposure was the development of incident breast cancer (ICD-10 code C50) considered as a time-varying exposure. Breast cancer was confirmed after the presence of a C50 code in more than 3 claims within a year or in an inpatient hospitalization claim. Treatments for breast cancer were identified from claims filled within 1 year after breast cancer diagnosis and used to classify patients as receiving only surgery or surgery plus chemotherapy, radiation therapy, or hormone treatment.

The study outcomes were the development of chronic diseases for which prior research indicates that breast cancer patients may be at increased risk, including common consequences of cancer treatments^12,38^. According to a recent systematic review, cure or control of cancer leads to a higher risk and earlier onset of multiple ageing-associated health problems resulting in chronic diseases^12^. Cancer treatment contribute to the ageing phenotypes of decreased resilience, geriatric syndromes and predisposition to age-related chronic diseases, including telomere attrition, cellular senescence, stem and progenitor cell dysfunction, macromolecular DNA damage and epigenetic alterations^12^. For this study, we initially included leukemia, endometrial cancer, myeloma, cardiomyopathy, osteoporosis, end stage renal disease (ESRD), pulmonary fibrosis, hair loss, muscular weakness, frailty, attention deficits, infertility, and hypothyroidism that breast cancer patients would experience due to premature of ageing due to breast cancer treatment. We then added hypertension, hyperlipidemia, and diabetes which could be important mediator or shared risk factors for the chronic diseases. However, we had to exclude hair loss, muscular weakness, frailty, attention deficits, and infertility as these conditions are difficult to define or to identify using claims data. Finally, we included leukemia, endometrial cancer, myeloma, cardiomyopathy, osteoporosis, end stage renal disease (ESRD), pulmonary fibrosis, hypothyroidism, type 2 diabetes, hypertension and hyperlipidemia. Leukemia (ICD-10 codes C91-C95), endometrial cancer (C54.1) and myeloma (C90) were confirmed after the presence of the same C code in more than 3 claims within a year or in an inpatient hospitalization claim^39^. Cardiomyopathy was defined as an inpatient hospitalization with a code I40-I43^40^. Osteoporosis was defined as the presence of codes M80-M82 in at least 2 claims with a bone mineral density examination code (HC341-HC345) within a year^39^. Hypothyroidism was defined as the presence of code E03 in 3 claims within a year or in an inpatient hospitalization claim^41^. Pulmonary fibrosis was defined as the presence of code E03 in more than 2 claims within a year^42^. ESRD was defined as a code N18, N19, Z49, Z940, Z992, or code V001, V003 with a hemodialysis code O7020, O9991, peritoneal dialysis code O7075, or kidney transplantation code R3280 in a claim^43^. Hypertension (I10-I13, I15), type 2 diabetes (E11–E14) and hyperlipidemia (E78) were defined as the presence of identical disease codes in at least two claims within a year, or the presence of a disease code claim and a code for a medication to control the disease in a prescription claim within a year. Outcomes were consider to occur at the time of the first claim with the diagnostic or medication code^12^.

Age and income level were obtained from the insurance eligibility database. Smoking habits (never smoker, ever smoker, and unknown), drinking habit s(none, moderate, heavy, and unknown), physical activity(none, 1–2 times per week, ≥ 3 times per week, and unknown), height, and weight were obtained from the health screening exam database. Body mass index (BMI) was calculated and categorized as underweight (< 18.5 kg/m^2^), normal (18.5–< 23 kg/m^2^), overweight (23–< 25 kg/m^2^), obese (≥ 25 kg/m^2^), and unknown. In addition, age at menarche and menopause status were obtained from the cancer screening exam. In terms of menopause status, we used menopause age from all of the screening during the study period and then coded whether the patients were pre- or post-menopause at the initial breast cancer screening comparing the current age and menopause age.

### Statistical analysis

Participants were included in the study on the date of their first health screening exam (baseline) and were followed-up until the development of a study endpoint, death, or the end of the study period (December 31, 2016). The study endpoints were the development of leukemia, endometrial cancer, myeloma, cardiomyopathy, osteoporosis, hypothyroidism, pulmonary fibrosis, ESRD, type 2 diabetes, hypertension, and hyperlipidemia. Each endpoint was analyzed separately, and for each end point analysis we included only participants who had not experienced the endpoint prior to the first screening exam. The number of participants included in the analysis will thus be different for each endpoint.

The study exposure was breast cancer development, considered as a time-varying variable. Participants who developed breast cancer contributed person-time to the exposed group from the time of breast cancer development. Unexposed person-time was contributed by participants who did not develop breast cancer and by participants who developed event prior to breast cancer development (Supplement Fig. 2). For each outcome, cases of breast cancer occurring after developing a study endpoint (outcome) were not included in the analysis as participant follow-up ended with the development of a study outcome. According to large population-based studies, the median time from diagnosis to breast cancer surgery was 27^44^. Since patients would receive various exam prior to surgery, to reduce the potential impact of surveillance bias, we considered that outcomes occurring in the first 31 days after a diagnosis of breast cancer corresponded to unexposed person-time.

Cumulative incidences and hazard rates were estimated the Kaplan–Meier method and a weighted kernel-density estimator using the estimated hazard contributions, respectively. We calculated hazard ratios (HR) with 95% confidence intervals (CI) for developing each chronic disease using a proportional hazards regression model with age as the time scale. To estimate age-dependent effects, we conducted time-varying analyses according to the split-age interval. Participants were assigned to the < 50 years age group until they reached the age of 50 years and to the age ≥ 50 years age group.

Follow-up time was classified as < 1, 1–< 3, 3–< 5, and ≥ 5 years from the time of breast cancer diagnosis. Because of the substantial overlap of treatment modalities (chemotherapy, radiation therapy, and hormone therapy), we used non-exclusive categories in the analysis of treatment modalities (for instance, the analysis of participants who received chemotherapy compared all participants who received chemotherapy to those who did not, irrespective of other treatment modalities).

To account for potential confounding factors at the health screening exam (baseline), we adjusted for body mass index, alcohol intake, moderate-vigorous physical activity, smoking status, income percentile, and for the presence of other comorbid conditions included in this study at the time of the health screening exam. When the variable had missing value, we considered as “none” for variables from the claim. On the other hand, for the variables from questionnaire in health screening data, we coded as missing category. Thus we adjusted for BMI, alcohol intake, physical activity, smoking status, income percentile, as well as for the baseline presence of comorbid conditions other than the corresponding outcome.

To account for competing risks due to mortality, we fitted a proportional subdistribution hazards regression model with death as the competing event^45^, with similar findings (not shown). We examined the proportional hazards assumption. All analyses were performed using STATA version 14 (StataCorp LP, College Station, TX, USA).

### Ethics approval and consent to participate

The Institutional Review Board (IRB) of the Samsung Medical Center approved this study (SMC-201912064) and waived the requirement for informed consent as we used only de-identified data.

## Supplementary Information

Supplementary Figure 1.

Supplementary Figure 2.

Supplementary Tables.

## Data Availability

Data that support the findings of this study are available from the National Health Insurance Sharing Service (https://nhiss.nhis.or.kr/) with the permission. Restrictions may apply due to the policy of the NHISS.
