# Supplementary material for: Temporal patterns of chronic disease incidence after breast cancer: a nationwide population-based cohort study
Source: Sci Rep. 2022 Mar 31;12:5489. doi: 10.1038/s41598-022-09542-w (PMC8971484; doi:10.1038/s41598-022-09542-w)
Supplement: Supplementary file 1 — Supplementary Figure 1. [file 41598_2022_9542_MOESM1_ESM.docx]

**Temporal patterns of chronic disease incidence after breast cancer:
A nationwide population-based cohort study**

Danbee Kang, PhD,^1,2*^ Minwoong Kang, MS,^2,3^* Yun Soo Hong, MD,^4^ Jihwan Park, MS,^4^ Jin Lee, MPH,^1,2^ Hwa Jeong Seo, PhD,^5^ Dong Wook Kim, PhD,^6^ Jin Seok Ahn, MD, PhD,^7^ Yeon Hee Park, MD, PhD,^1,7^ Se Kyung Lee, MD, PhD,^8^ Dong Wook Shin, MD, PhD,^1,3,9^ Eliseo Guallar, MD, DrPH,^2,4^ Juhee Cho, PhD,^1,2,3,4†^

**
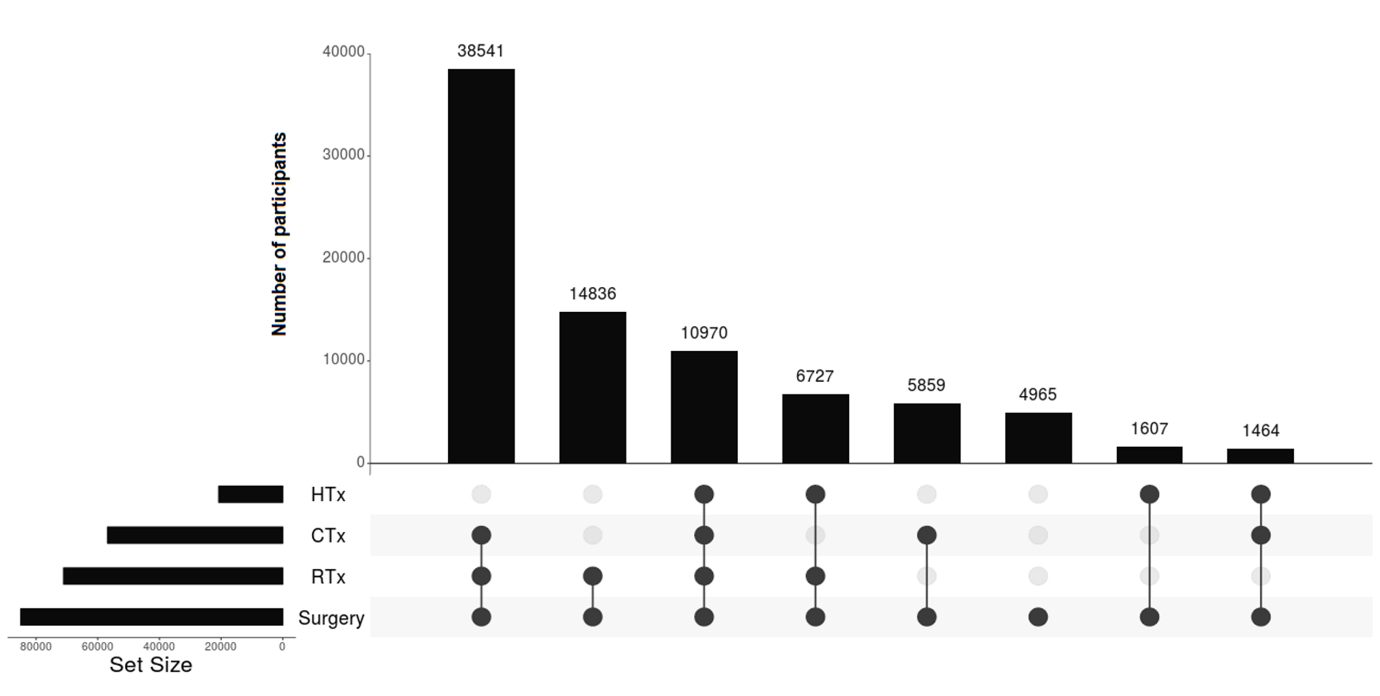
**

**Supplement Figure 1.** UpSet plot of treatment combinations in breast cancer patients.

The vertical bars reflect how many participants received a specific combination of treatment modalities, identified by the dots below each bar. For instance, the first vertical bar indicates that 38,541 participants received surgery plus radiation therapy plus chemotherapy but no hormone therapy. The second vertical bar indicates that 14,836 participants receiver surgery plus radiation therapy, but no chemotherapy or hormone therapy. The horizontal bars reflect how many participants received each treatment modality across all treatment combinations.

CTx, chemotherapy; HTx, hormone therapy; RTx, radiation therapy.
