# Supplementary material for: Temporal patterns of chronic disease incidence after breast cancer: a nationwide population-based cohort study
Source: Sci Rep. 2022 Mar 31;12:5489. doi: 10.1038/s41598-022-09542-w (PMC8971484; doi:10.1038/s41598-022-09542-w)
Supplement: Supplementary file 2 — Supplementary Figure 2. [file 41598_2022_9542_MOESM2_ESM.docx]

**Temporal patterns of chronic disease incidence after breast cancer:
A nationwide population-based cohort study**

Danbee Kang, PhD,^1,2*^ Minwoong Kang, MS,^2,3^* Yun Soo Hong, MD,^4^ Jihwan Park, MS,^4^ Jin Lee, MPH,^1,2^ Hwa Jeong Seo, PhD,^5^ Dong Wook Kim, PhD,^6^ Jin Seok Ahn, MD, PhD,^7^ Yeon Hee Park, MD, PhD,^1,7^ Se Kyung Lee, MD, PhD,^8^ Dong Wook Shin, MD, PhD,^1,3,9^ Eliseo Guallar, MD, DrPH,^2,4^ Juhee Cho, PhD,^1,2,3,4†^

**
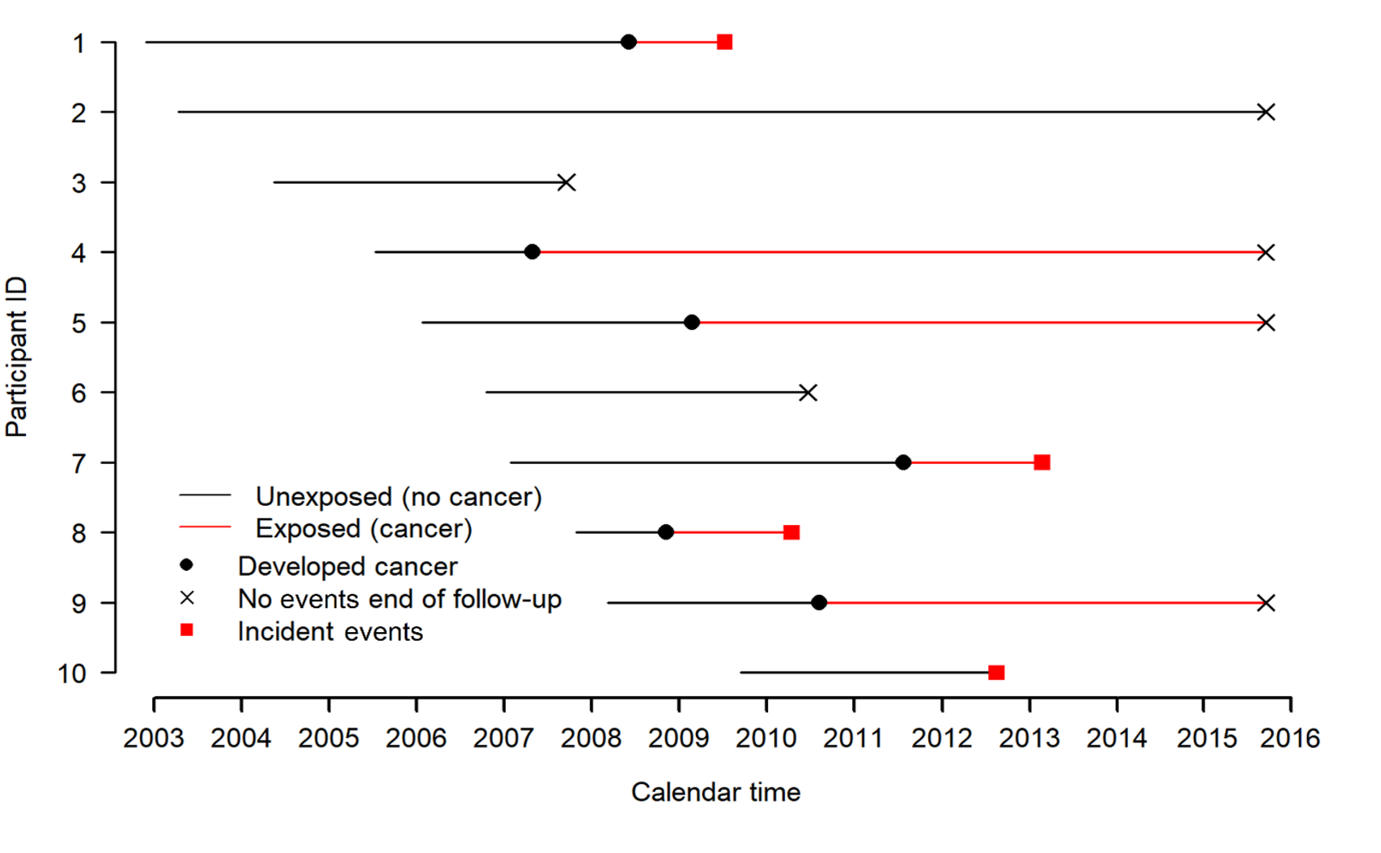
**

**Supplement Figure 2.** The statistical concept of the study
