# Supplementary material for: Temporal patterns of chronic disease incidence after breast cancer: a nationwide population-based cohort study
Source: Sci Rep. 2022 Mar 31;12:5489. doi: 10.1038/s41598-022-09542-w (PMC8971484; doi:10.1038/s41598-022-09542-w)
Supplement: Supplementary file 3 — Supplementary Tables. [file 41598_2022_9542_MOESM3_ESM.docx]

**Temporal patterns of chronic disease incidence after breast cancer:
A nationwide population-based cohort study**

Danbee Kang, PhD,^1,2*^ Minwoong Kang, MS,^2,3^* Yun Soo Hong, MD,^4^ Jihwan Park, MS,^4^ Jin Lee, MPH,^1,2^ Hwa Jeong Seo, PhD,^5^ Dong Wook Kim, PhD,^6^ Jin Seok Ahn, MD, PhD,^7^ Yeon Hee Park, MD, PhD,^1,7^ Se Kyung Lee, MD, PhD,^8^ Dong Wook Shin, MD, PhD,^1,3,9^ Eliseo Guallar, MD, DrPH,^2,4^ Juhee Cho, PhD,^1,2,3,4†^

**Supplement Table 1.** Hazard ratios (95% confidence intervals) for incident comorbidity associated with incident breast cancer by age.

| **Outcome** | **Age <50** | **Age ≥50** | **P for interaction** |
| --- | --- | --- | --- |
|  | **HR (95% CI)** | **HR (95% CI)** |  |
| Leukemia (N = 1,142,643) | 5.51 (3.86, 7.86) | 3.83 (3.07, 4.78) | 0.09 |
| Cardiomyopathy (N = 1,142,076) | 10.68 (7.44, 15.35) | 2.56 (2.09, 3.15) | < 0.01 |
| Osteoporosis (N = 1,070,659) | 9.63 (9.21, 10.07) | 2.29 (2.23, 2.35) | < 0.01 |
| Endometrial cancer (N = 1,142,643) | 2.51 (1.80, 3.50) | 2.76 (2.32, 3.29) | 0.62 |
| Hypothyroidism (N = 1,122,284) | 2.16 (2.03, 2.29) | 1.50 (1.43, 1.57) | < 0.01 |
| Pulmonary fibrosis (N = 1,142,493) | 4.29 (2.44, 7.53) | 1.25 (0.86, 1.81) | < 0.01 |
| Myeloma (N = 1,142,643) | 4.10 (1.61, 10.48) | 1.38 (0.92, 2.05) | 0.04 |
| Hyperlipidemia (N = 1,054,439) | 2.20 (2.14, 2.27) | 1.18 (1.16, 1.21) | < 0.01 |
| End-stage renal disease (N = 1,141,344) | 1.02 (0.52, 1.97) | 1.20 (0.95, 1.51) | 0.64 |
| Type 2 diabetes (N = 1,070,608) | 1.72 (1.62, 1.82) | 1.02 (0.99, 1.06) | < 0.01 |
| Hypertension (N = 974,915) | 0.70 (0.66, 0.74) | 0.58 (0.56, 0.60) | < 0.01 |

Each outcome was analyzed separately. Each analysis was performed among participants free of the outcome disease at baseline and adjusted for body mass index category (underweight, normal, overweight, obese, and unknown), alcohol intake (none, moderate, heavy, and unknown), physical activity (none, 1-2 times per week, ≥3 times per week, and unknown), smoking status (never smoker, ever smoker, and unknown), income percentile (Medical Aid, ≤30^th^, 31^st^–70^th^, >70^th^ percentile), as well as for the baseline presence of comorbid conditions other than the corresponding outcome.

**Supplement Table 2.** Hazard ratios (95% confidence intervals) for incident outcomes after incident breast cancer by breast cancer treatment.

| **Outcome*** | **Surgery only** | **Surgery plus chemotherapy, radiation therapy, or hormone therapy** | **Surgery plus chemotherapy** | **Surgery plus radiation therapy** | **Surgery plus hormone therapy** |
| --- | --- | --- | --- | --- | --- |
| Leukemia (N = 1,142,643) | 0.39 (0.06, 2.80) | 4.54 (3.76, 5.48) | 5.09 (4.13, 6.26) | 4.77 (3.91, 5.83) | 10.55 (8.33, 13.34) |
| Cardiomyopathy (N = 1,142,076) | 0.85 (0.32, 2.28) | 3.57 (2.99, 4.27) | 5.00 (4.16, 6.02) | 3.68 (3.04, 4.46) | 7.23 (5.70, 9.17) |
| Osteoporosis (N = 1,070,659) | 2.41 (2.21, 2.63) | 3.04 (2.97, 3.12) | 3.15 (3.06, 3.23) | 3.09 (3.02, 3.17) | 3.08 (2.94, 3.22) |
| Endometrial cancer (N = 1,142,643) | 3.02 (1.75, 5.22) | 2.68 (2.28, 3.15) | 2.69 (2.24, 3.23) | 2.44 (2.03, 2.93) | 4.51 (3.55, 5.73) |
| Hypothyroidism (N = 1,122,284) | 1.35 (1.15, 1.58) | 1.72 (1.65, 1.78) | 1.83 (1.75, 1.91) | 1.77 (1.70, 1.84) | 1.91 (1.78, 2.05) |
| Pulmonary fibrosis (N = 1,142,493) | 2.31 (1.03, 5.18) | 1.55 (1.11, 2.15) | 1.61 (1.09, 2.37) | 1.64 (1.15, 2.33) | 2.07 (1.20, 3.59) |
| Myeloma (N = 1,142,643) | 1.58 (0.51, 4.92) | 1.54 (1.05, 2.26) | 1.52 (0.96, 2.40) | 1.41 (0.91, 2.18) | 3.63 (2.21, 5.99) |
| Hyperlipidemia (N = 1,054,439) | 1.08 (1.01, 1.16) | 1.39 (1.37, 1.41) | 1.38 (1.35, 1.41) | 1.46 (1.44, 1.49) | 1.37 (1.33, 1.41) |
| End-stage renal disease (N = 1,141,344) | 0.37 (0.12, 1.16) | 1.22 (0.98, 1.52) | 1.02 (0.76, 1.36) | 1.20 (0.94, 1.53) | 2.80 (2.11, 3.70) |
| Type 2 diabetes (N = 1,070,608) | 1.02 (0.91, 1.13) | 1.14 (1.10, 1.17) | 1.17 (1.14, 1.22) | 1.13 (1.10, 1.17) | 1.33 (1.26, 1.40) |
| Hypertension (N = 974,915) | 0.79 (0.72, 0.87) | 0.60 (0.58, 0.61) | 0.58 (0.56, 0.60) | 0.57 (0.55, 0.59) | 0.75 (0.71, 0.79) |

Each outcome was analyzed separately. Each analysis was performed among participants free of the outcome disease at baseline and adjusted for body mass index category (underweight, normal, overweight, obese, and unknown), alcohol intake (none, moderate, heavy, and unknown), physical activity (none, 1-2 times per week, ≥3 times per week, and unknown), smoking status (never smoker, ever smoker, and unknown), income percentile (Medical Aid, ≤30^th^, 31^st^–70^th^, >70^th^ percentile), as well as for the baseline presence of comorbid conditions other than the corresponding outcome. The reference group for hazard ratios was no breast cancer. The groups surgery only and surgery plus chemotherapy, radiation therapy, or hormone therapy are mutually exclusive. The groups surgery plus chemotherapy, surgery plus radiation therapy, and surgery plus hormone therapy are not mutually exclusive.

**Supplement Table 3.** Hazard ratios (95% confidence intervals) for incident outcomes after incident breast cancer by time after breast cancer diagnosis in patient with and without chemotherapy, radiation therapy, or hormone therapy.

| **Comorbidity*** | | **No breast cancer** | **< 1 year after**  **diagnosis** | **1-<3 years after diagnosis** | **3-<5 years after diagnosis** | **≥5 years after diagnosis** |
| --- | --- | --- | --- | --- | --- | --- |
|  |  | **HR (95% CI)** | **HR (95% CI)** | **HR (95% CI)** | **HR (95% CI)** | **HR (95% CI)** |
| **Chemotherapy** | | | |  |  |  |
|  | Leukemia (N = 1,142,643) | *Reference* | 3.18 (1.75, 5.76) | 6.98 (5.11, 9.53) | 7.35 (5.18, 10.43) | 2.63 (1.60, 4.32) |
|  | Cardiomyopathy (N = 1,142,076) | *Reference* | 7.02 (4.82, 10.23) | 5.78 (4.24, 7.87) | 4.63 (3.20, 6.88) | 3.62 (2.53, 5.18) |
|  | Osteoporosis (N = 1,070,659) | *Reference* | 4.61 (4.38, 4.85) | 5.01 (4.83, 5.21) | 2.53 (2.39, 2.69) | 0.91 (0.84, 0.99) |
|  | Endometrial cancer (N = 1,142,643) | *Reference* | 1.72 (1.01, 2.91) | 1.49 (0.97, 2.29) | 3.45 (2.48, 4.81) | 3.96 (3.01, 5.22) |
|  | Hypothyroidism (N = 1,122,284) | *Reference* | 2.22 (2.03, 2.42) | 2.32 (2.17, 2.48) | 1.43 (1.30, 1.59) | 1.31 (1.19, 1.45) |
|  | Pulmonary fibrosis (N = 1,142,493) | *Reference* | 1.53 (0.57, 4.08) | 1.68 (0.84, 3.37) | 0.53 (0.13, 2.12) | 2.32 (1.34, 4.02) |
|  | Myeloma (N = 1,142,643) | *Reference* | 2.12 (0.79, 5.69) | 0.86 (0.28, 2.69) | 1.07 (0.34, 3.32) | 2.06 (1.07, 3.99) |
|  | Hyperlipidemia (N = 1,054,439) | *Reference* | 1.61 (1.54, 1.67) | 1.52 (1.47, 1.57) | 1.25 (1.20, 1.31) | 1.19 (1.15, 1.24) |
|  | ESRD (N = 1,141,344) | *Reference* | 0.99 (0.49, 1.97) | 1.14 (0.69, 1.86) | 0.67 (0.32, 1.41) | 1.19 (0.73, 1.94) |
|  | Type 2 diabetes (N = 1,070,608) | *Reference* | 1.20 (1.88, 2.12) | 1.15 (1.08, 1.23) | 0.92 (0.85, 1.00) | 0.91 (0.85, 0.98) |
|  | Hypertension (N = 974,915) | *Reference* | 0.71 (0.66, 0.77) | 0.55 (0.52, 0.59) | 0.49 (0.45, 0.57) | 0.59 (0.56, 0.63) |
| **Radiation therapy** | |  |  |  |  |  |
|  | Leukemia (N = 1,142,643) | *Reference* | 2.85 (1.65, 4.94) | 5.54 (4.06, 7.57) | 6.42 (4.55, 9.06) | 3.81 (2.52, 5.78) |
|  | Cardiomyopathy (N = 1,142,076) | *Reference* | 4.85 (3.33, 7.07) | 4.00 (2.92, 5.49) | 3.56 (2.34, 5.25) | 2.64 (1.74, 3.99) |
|  | Osteoporosis (N = 1,070,659) | *Reference* | 4.32 (4.13, 4.52) | 4.67 (4.50, 4.83) | 2.24 (2.10, 2.38) | 0.86 (0.79, 0.94) |
|  | Endometrial cancer (N = 1,142,643) | *Reference* | 1.78 (1.12, 2.84) | 1.66 (1.14, 2.42) | 3.41 (2.49, 4.67) | 3.08 (2.25, 4.23) |
|  | Hypothyroidism (N = 1,122,284) | *Reference* | 2.14 (1.97, 2.32) | 2.17 (2.03, 2.31) | 1.34 (1.21, 1.47) | 1.29 (1.17, 1.42) |
|  | Pulmonary fibrosis (N = 1,142,493) | *Reference* | 1.92 (0.91, 4.04) | 1.43 (0.74, 2.76) | 0.87 (0.32, 2.31) | 2.33 (1.34, 4.04) |
|  | Myeloma (N = 1,142,643) | *Reference* | 1.91 (0.79, 4.62) | 0.88 (0.33, 2.34) | 1.17 (0.44, 3.13) | 1.85 (0.92, 3.73) |
|  | Hyperlipidemia (N = 1,054,439) | *Reference* | 1.68 (1.62, 1.74) | 1.57 (1.52, 1.62) | 1.35 (1.30, 1.40) | 1.26 (1.22, 1.33) |
|  | ESRD (N = 1,141,344) | *Reference* | 0.90 (0.48, 1.66) | 1.40 (0.95, 2.06) | 1.01 (0.59, 1.75) | 1.35 (0.84, 2.15) |
|  | Type 2 diabetes (N = 1,070,608) | *Reference* | 1.72 (1.62, 1.82) | 1.12 (1.06, 1.19) | 0.92 (0.86, 0.99) | 0.89 (0.83, 0.96) |
|  | Hypertension (N = 974,915) | *Reference* | 0.71 (0.66, 0.75) | 0.56 (0.53, 0.59) | 0.49 (0.46, 0.53) | 0.56 (0.53, 0.60) |
| **Hormone therapy** | |  |  |  |  |  |
|  | Leukemia (N = 1,142,643) | *Reference* | 3.12 (1.17, 8.35) | 13.36 (9.27, 19.26) | 17.04 (11.68, 24.87) | 7.03 (4.21, 11.73) |
|  | Cardiomyopathy (N = 1,142,076) | *Reference* | 11.14 (6.98, 17.76) | 7.84 (5.18, 15.18) | 7.92 (4.97, 12.63) | 4.26 (2.51, 7.23) |
|  | Osteoporosis (N = 1,070,659) | *Reference* | 4.52 (4.15, 4.93) | 4.81 (4.51, 5.12) | 2.45 (2.2, 2.72) | 0.84 (0.71, 0.98) |
|  | Endometrial cancer (N = 1,142,643) | *Reference* | 2.91 (1.45, 5.83) | 3.72 (2.34, 5.92) | 5.61 (3.61, 8.73) | 5.50 (3.67, 8.23) |
|  | Hypothyroidism (N = 1,122,284) | *Reference* | 2.42 (2.10, 2.79) | 2.15 (1.91, 2.41) | 1.79 (1.45, 1.98) | 1.42 (1.21, 1.67) |
|  | Pulmonary fibrosis (N = 1,142,493) | *Reference* | 1.99 (0.50, 7.98) | 2.19 (0.82, 5.85) | 1.41 (0.35, 5.64) | 2.48 (1.03, 5.99) |
|  | Myeloma (N = 1,142,643) | *Reference* | 5.92 (2.21, 15.86) | 2.40 (0.77, 7.48) | 5.03 (2.08, 12.16) | 2.69 (1.00, 7.21) |
|  | Hyperlipidemia (N = 1,054,439) | *Reference* | 1.56 (1.45, 1.67) | 1.41 (1.33, 1.49) | 1.34 (1.25, 1.69) | 1.23 (1.16, 1.31) |
|  | ESRD (N = 1,141,344) | *Reference* | 2.18 (1.04, 5.10) | 3.21 (2.01, 5.10) | 3.20 (1.85 5.53) | 2.40 (1.36, 3.24) |
|  | Type 2 diabetes (N = 1,070,608) | *Reference* | 1.98 (1.78, 2.20) | 1.36 (1.24, 1.50) | 1.16 (1.03, 1.31) | 1.02 (0.91, 1.15) |
|  | Hypertension (N = 974,915) | *Reference* | 1.05 (0.95, 1.71) | 0.79 (0.72, 0.86) | 0.58 (0.51, 0.65) | 0.65 (0.58, 0.72) |

ESRD, End-stage renal disease

Each outcome was analyzed separately. Each analysis was performed among participants free of the outcome disease at baseline and adjusted for body mass index category (underweight, normal, overweight, obese, and unknown), alcohol intake (none, moderate, heavy, and unknown), physical activity (none, 1-2 times per week, ≥3 times per week, and unknown), smoking status (never smoker, ever smoker, and unknown), income percentile (Medical Aid, ≤30^th^, 31^st^–70^th^, >70^th^ percentile), as well as for the baseline presence of comorbid conditions other than the corresponding outcome. The reference group for hazard ratios was no breast cancer. The groups surgery only and surgery plus chemotherapy, radiation therapy, or hormone therapy are mutually exclusive.
